# Supplementary material for: Breakdown of local information processing may underlie isoflurane anesthesia effects
Source: PLoS Comput Biol. 2017 Jun 1;13(6):e1005511. doi: 10.1371/journal.pcbi.1005511 (PMC5453425; doi:10.1371/journal.pcbi.1005511)
Supplement: S1 Table — R˜2 and max(R2) indicate the median and maximum of R2 over recordings per condition, respectively. (PDF) [file pcbi.1005511.s002.pdf]

**animal**	**isoflurane level**	**recording site**	p	$\widetilde{R}^2$ $(max(R^2)$
1	iso 0.0 %	PFC	1.0000	0.024 (0.289)
		V1	0.1028	0.003 (0.153)
	iso 0.5 %	PFC	0.0013**	0.001 (0.122)
		V1	0.0000***	0.014 (0.114)
	iso 1.0 %	PFC	0.0000***	0.005 (0.271)
		V1	0.9998	0.004 (0.051)
2	iso 0.0 %	PFC	0.0000***	0.022 (0.116)
		V1	0.0000***	0.029 (0.175)
	iso 0.5 %	PFC	0.7672	0.007 (0.018)
		V1	0.0000***	0.017 (0.076)
	iso 1.0 %	PFC	0.0000***	0.008 (0.121)
		V1	0.8247	0.009 (0.214)

$^*p < 0.05$; $^{**}p < 0.01$; $^{***}p < 0.001$
