# Supplementary material for: Breakdown of local information processing may underlie isoflurane anesthesia effects
Source: PLoS Comput Biol. 2017 Jun 1;13(6):e1005511. doi: 10.1371/journal.pcbi.1005511 (PMC5453425; doi:10.1371/journal.pcbi.1005511)
Supplement: S2 Table — R˜2 and max(R2) indicate the median and maximum of R2 over recordings per condition, respectively. (PDF) [file pcbi.1005511.s003.pdf]

**animal**	**isoflurane level**	**recording site**	p	$\widetilde{R}^2$ $(max(R^2)$
1	iso 0.0 %	PFC	1.0000	0.087 (0.705)
		V1	1.0000	0.079 (0.452)
	iso 0.5 %	PFC	1.0000	0.036 (0.093)
		V1	1.0000	0.062 (0.217)
	iso 1.0 %	PFC	1.0000	0.010 (0.474)
		V1	1.0000	0.018 (0.083)
2	iso 0.0 %	PFC	1.0000	0.042 (0.699)
		V1	1.0000	0.162 (0.363)
	iso 0.5 %	PFC	1.0000	0.041 (0.130)
		V1	1.0000	0.031 (0.289)
	iso 1.0 %	PFC	1.0000	0.118 (0.602)
		V1	1.0000	0.018 (0.578)

$^{*}p < 0.05$; $^{**}p < 0.01$; $^{***}p < 0.001$
