# Supplementary material for: Breakdown of local information processing may underlie isoflurane anesthesia effects
Source: PLoS Comput Biol. 2017 Jun 1;13(6):e1005511. doi: 10.1371/journal.pcbi.1005511 (PMC5453425; doi:10.1371/journal.pcbi.1005511)
Supplement: S3 Table — R˜2 and max(R2) indicate the median and maximum of R2 over recordings per condition, respectively. (PDF) [file pcbi.1005511.s004.pdf]

**animal**	**isoflurane level**	**direction**	ACT		signal variance	
			p	$\widetilde{R}^2$ $(max(R^2)$	p	$\widetilde{R}^2$ $(max(R^2)$
1	iso 0.0 %	PFC → V1	1.0000	0.035 (0.110)	0.9861	0.009 (0.060)
		V1 → PFC	0.9996	0.029 (0.215)	0.6644	0.024 (0.089)
	iso 0.5 %	PFC → V1	1.0000	0.016 (0.110)	1.0000	0.024 (0.033)
		V1 → PFC	0.7310	0.003 (0.014)	0.0063*	0.005 (0.011)
	iso 1.0 %	PFC → V1	1.0000	0.006 (0.047)	0.5836	0.002 (0.037)
		V1 → PFC	0.0069*	0.001 (0.032)	0.0000***	0.005 (0.092)
2	iso 0.0 %	PFC → V1	1.0000	0.063 (0.105)	0.8315	0.008 (0.025)
		V1 → PFC	0.9996	0.006 (0.041)	0.0434	0.005 (0.031)
	iso 0.5 %	PFC → V1	1.0000	0.115 (0.155)	0.0440	0.017 (0.103)
		V1 → PFC	1.0000	0.022 (0.125)	0.9985	0.005 (0.086)
	iso 1.0 %	PFC → V1	0.0000**	0.011 (0.111)	0.0000***	0.012 (0.058)
		V1 → PFC	0.0076*	0.009 (0.031)	0.0000***	0.015 (0.032)

*$p < 0.05$; **$p < 0.01$; ***$p < 0.001$
