# Supplementary material for: Breakdown of local information processing may underlie isoflurane anesthesia effects
Source: PLoS Comput Biol. 2017 Jun 1;13(6):e1005511. doi: 10.1371/journal.pcbi.1005511 (PMC5453425; doi:10.1371/journal.pcbi.1005511)
Supplement: S4 Table — R˜2 and max(R2) indicate the median and maximum of R2 over recordings per condition, respectively. (PDF) [file pcbi.1005511.s005.pdf]

| **animal** | **level** | **site** | | $\delta$ | | $\theta$ | | $\alpha$ |
|---|---|---|---|---|---|---|---|---|
| | | | $p$ | $\widetilde{R}^2$ $(max(R^2)$ | $p$ | $\widetilde{R}^2$ $(max(R^2)$ | $p$ | $\widetilde{R}^2$ $(max(R^2)$ |
| 1 | iso 0.0 % | PFC | 1.0000 | 0.068 (0.418) | 0.0000*** | 0.021 (0.201) | 0.0000*** | 0.022 (0.258) |
| | | V1 | 0.0000*** | 0.041 (0.275) | 0.0003** | 0.020 (0.160) | 0.0000*** | 0.029 (0.166) |
| | iso 0.5 % | PFC | 0.0000*** | 0.019 (0.174) | 0.0000*** | 0.005 (0.040) | 0.0000*** | 0.003 (0.075) |
| | | V1 | 0.0000*** | 0.008 (0.129) | 0.0093 | 0.024 (0.060) | 0.0986 | 0.009 (0.090) |
| | iso 1.0 % | PFC | 0.0000*** | 0.006 (0.036) | 0.8710 | 0.005 (0.093) | 0.0445 | 0.003 (0.028) |
| | | V1 | 0.2751 | 0.010 (0.303) | 0.0000*** | 0.002 (0.054) | 0.0000*** | 0.002 (0.030) |
| 2 | iso 0.0 % | PFC | 0.0000*** | 0.158 (0.359) | 0.0000*** | 0.044 (0.089) | 0.0000*** | 0.025 (0.075) |
| | | V1 | 0.0000*** | 0.005 (0.303) | 0.0000*** | 0.023 (0.228) | 0.0000*** | 0.042 (0.238) |
| | iso 0.5 % | PFC | 0.0000*** | 0.019 (0.255) | 0.0000*** | 0.007 (0.018) | 0.0000*** | 0.006 (0.074) |
| | | V1 | 0.0000*** | 0.025 (0.098) | 0.6476 | 0.024 (0.068) | 1.0000 | 0.017 (0.045) |
| | iso 1.0 % | PFC | 0.0000*** | 0.005 (0.298) | 0.9729 | 0.005 (0.021) | 0.9999 | 0.004 (0.151) |
| | | V1 | 0.0063* | 0.043 (0.302) | 0.7462 | 0.001 (0.039) | 1.0000 | 0.003 (0.051) |

| **animal** | **level** | **site** | | $\beta$ | | $\gamma$ |
|---|---|---|---|---|---|---|
| | | | $p$ | $\widetilde{R}^2$ $(max(R^2)$ | $p$ | $\widetilde{R}^2$ $(max(R^2)$ |
| 1 | iso 0.0 % | PFC | 0.1179 | 0.016 (0.154) | 1.0000 | 0.036 (0.089) |
| | | V1 | 0.0001*** | 0.039 (0.163) | 1.0000 | 0.018 (0.331) |
| | iso 0.5 % | PFC | 0.0000*** | 0.003 (0.023) | 0.5147 | 0.007 (0.068) |
| | | V1 | 0.9981 | 0.025 (0.081) | 1.0000 | 0.005 (0.009) |
| | iso 1.0 % | PFC | 0.7781 | 0.001 (0.044) | 0.9392 | 0.004 (0.063) |
| | | V1 | 0.1122 | 0.003 (0.045) | 0.8076 | 0.003 (0.061) |
| 2 | iso 0.0 % | PFC | 0.0000*** | 0.005 (0.047) | 1.0000 | 0.006 (0.077) |
| | | V1 | 0.2450 | 0.015 (0.185) | 1.0000 | 0.027 (0.060) |
| | iso 0.5 % | PFC | 0.0000*** | 0.007 (0.094) | 0.0000*** | 0.014 (0.088) |
| | | V1 | 1.0000 | 0.036 (0.087) | 1.0000 | 0.010 (0.080) |
| | iso 1.0 % | PFC | 1.0000 | 0.007 (0.073) | 1.0000 | 0.008 (0.227) |
| | | V1 | 1.0000 | 0.001 (0.062) | 1.0000 | 0.017 (0.147) |

$^{*}p < 0.05$; $^{**}p < 0.01$; $^{***}p < 0.001$
