# Supplementary material for: Breakdown of local information processing may underlie isoflurane anesthesia effects
Source: PLoS Comput Biol. 2017 Jun 1;13(6):e1005511. doi: 10.1371/journal.pcbi.1005511 (PMC5453425; doi:10.1371/journal.pcbi.1005511)
Supplement: S5 Table — R˜2 and max(R2) indicate the median and maximum of R2 over recordings per condition, respectively. (PDF) [file pcbi.1005511.s006.pdf]

**animal**	**level**	**site**	δ		θ		α	
			p	$\widetilde{R}^2$ $(max(R^2)$	p	$\widetilde{R}^2$ $(max(R^2)$	p	$\widetilde{R}^2$ $(max(R^2)$
1	iso 0.0 %	PFC	1.0000	0.095 (0.458)	1.0000	0.015 (0.201)	1.0000	0.020 (0.271)
		V1	1.0000	0.091 (0.706)	0.9998	0.037 (0.109)	1.0000	0.041 (0.097)
	iso 0.5 %	PFC	1.0000	0.046 (0.202)	1.0000	0.004 (0.036)	1.0000	0.003 (0.059)
		V1	1.0000	0.035 (0.133)	0.9100	0.040 (0.051)	0.8029	0.002 (0.086)
	iso 1.0 %	PFC	1.0000	0.020 (0.079)	0.0359	0.005 (0.082)	0.9198	0.004 (0.023)
		V1	1.0000	0.010 (0.461)	0.9941	0.003 (0.061)	0.9991	0.004 (0.032)
2	iso 0.0 %	PFC	1.0000	0.171 (0.395)	1.0000	0.058 (0.110)	1.0000	0.024 (0.060)
		V1	1.0000	0.031 (0.723)	1.0000	0.036 (0.135)	1.0000	0.048 (0.170)
	iso 0.5 %	PFC	1.0000	0.035 (0.298)	1.0000	0.011 (0.016)	1.0000	0.015 (0.088)
		V1	1.0000	0.038 (0.135)	0.0428	0.021 (0.073)	0.0000***	0.016 (0.045)
	iso 1.0 %	PFC	1.0000	0.015 (0.580)	0.0000***	0.006 (0.076)	0.0000***	0.008 (0.219)
		V1	1.0000	0.112 (0.601)	0.1329	0.003 (0.062)	0.0000***	0.009 (0.094)

**animal**	**level**	**site**	β		γ	
			p	$\widetilde{R}^2$ $(max(R^2)$	p	$\widetilde{R}^2$ $(max(R^2)$
1	iso 0.0 %	PFC	0.9665	0.018 (0.165)	0.0000***	0.052 (0.097)
		V1	0.9999	0.046 (0.121)	0.0000***	0.014 (0.358)
	iso 0.5 %	PFC	1.0000	0.007 (0.020)	0.5510	0.013 (0.064)
		V1	0.0000***	0.028 (0.089)	0.0000***	0.006 (0.011)
	iso 1.0 %	PFC	0.2144	0.005 (0.056)	0.0269	0.006 (0.082)
		V1	0.4394	0.002 (0.040)	0.2250	0.003 (0.053)
2	iso 0.0 %	PFC	1.0000	0.011 (0.043)	0.0000***	0.001 (0.068)
		V1	0.5631	0.022 (0.094)	0.0000***	0.037 (0.061)
	iso 0.5 %	PFC	1.0000	0.013 (0.107)	1.0000	0.012 (0.095)
		V1	0.0000***	0.034 (0.096)	0.0000***	0.010 (0.102)
	iso 1.0 %	PFC	0.0000***	0.007 (0.051)	0.0000***	0.012 (0.290)
		V1	0.0017*	0.011 (0.072)	0.0000***	0.013 (0.103)

$^{*}p < 0.05$; $^{**}p < 0.01$; $^{***}p < 0.001$
