# Supplementary material for: Breakdown of local information processing may underlie isoflurane anesthesia effects
Source: PLoS Comput Biol. 2017 Jun 1;13(6):e1005511. doi: 10.1371/journal.pcbi.1005511 (PMC5453425; doi:10.1371/journal.pcbi.1005511)
Supplement: S6 Table — R˜2 and max(R2) indicate the median and maximum of R2 over recordings per condition, respectively. (PDF) [file pcbi.1005511.s007.pdf]

| **animal** | **level** | **direction** | $\delta$ | | $\theta$ | | $\alpha$ | |
|---|---|---|---|---|---|---|---|---|
| | | | $p$ | $\widetilde{R}^2$ $(max(R^2)$ | $p$ | $\widetilde{R}^2$ $(max(R^2)$ | $p$ | $\widetilde{R}^2$ $(max(R^2)$ |
| 1 | iso 0.0 % | PFC → V1 | 1.0000 | 0.024 (0.101) | 1.0000 | 0.034 (0.122) | 1.0000 | 0.061 (0.146) |
| | | V1 → PFC | 0.9662 | 0.030 (0.230) | 0.8389 | 0.009 (0.045) | 1.0000 | 0.008 (0.047) |
| | iso 0.5 % | PFC → V1 | 1.0000 | 0.033 (0.090) | 1.0000 | 0.073 (0.132) | 1.0000 | 0.013 (0.117) |
| | | V1 → PFC | 0.1319 | 0.001 (0.010) | 1.0000 | 0.018 (0.058) | 0.9097 | 0.001 (0.015) |
| | iso 1.0 % | PFC → V1 | 1.0000 | 0.009 (0.058) | 1.0000 | 0.009 (0.029) | 1.0000 | 0.012 (0.038) |
| | | V1 → PFC | 0.0947 | 0.002 (0.024) | 1.0000 | 0.003 (0.116) | 0.9996 | 0.004 (0.058) |
| 2 | iso 0.0 % | PFC → V1 | 1.0000 | 0.060 (0.102) | 1.0000 | 0.055 (0.121) | 1.0000 | 0.036 (0.052) |
| | | V1 → PFC | 0.9998 | 0.006 (0.038) | 1.0000 | 0.012 (0.056) | 0.9998 | 0.010 (0.040) |
| | iso 0.5 % | PFC → V1 | 1.0000 | 0.108 (0.157) | 1.0000 | 0.115 (0.166) | 1.0000 | 0.079 (0.160) |
| | | V1 → PFC | 1.0000 | 0.026 (0.149) | 1.0000 | 0.015 (0.052) | 0.6675 | 0.003 (0.009) |
| | iso 1.0 % | PFC → V1 | 0.0000*** | 0.009 (0.095) | 1.0000 | 0.010 (0.037) | 1.0000 | 0.007 (0.030) |
| | | V1 → PFC | 0.0153 | 0.008 (0.023) | 1.0000 | 0.006 (0.035) | 0.7369 | 0.001 (0.017) |

| **animal** | **level** | **site** | $\beta$ | | $\gamma$ | |
|---|---|---|---|---|---|---|
| | | | $p$ | $\widetilde{R}^2$ $(max(R^2)$ | $p$ | $\widetilde{R}^2$ $(max(R^2)$ |
| 1 | iso 0.0 % | PFC → V1 | 1.0000 | 0.069 (0.157) | 0.0352 | 0.005 (0.044) |
| | | V1 → PFC | 0.9612 | 0.010 (0.040) | 0.0009** | 0.010 (0.028) |
| | iso 0.5 % | PFC → V1 | 1.0000 | 0.033 (0.121) | 0.0611 | 0.031 (0.038) |
| | | V1 → PFC | 0.9991 | 0.010 (0.039) | 0.0014** | 0.021 (0.028) |
| | iso 1.0 % | PFC → V1 | 0.0000*** | 0.004 (0.019) | 0.0000*** | 0.007 (0.054) |
| | | V1 → PFC | 0.0912 | 0.002 (0.063) | 0.0000*** | 0.006 (0.109) |
| 2 | iso 0.0 % | PFC → V1 | 1.0000 | 0.021 (0.040) | 0.3062 | 0.015 (0.036) |
| | | V1 → PFC | 0.9998 | 0.002 (0.039) | 0.2046 | 0.002 (0.041) |
| | iso 0.5 % | PFC → V1 | 1.0000 | 0.107 (0.132) | 0.6054 | 0.006 (0.013) |
| | | V1 → PFC | 0.0186 | 0.004 (0.019) | 0.0000*** | 0.022 (0.041) |
| | iso 1.0 % | PFC → V1 | 0.9940 | 0.004 (0.039) | 0.0025* | 0.003 (0.027) |
| | | V1 → PFC | 0.9122 | 0.002 (0.013) | 0.0000*** | 0.002 (0.058) |

$^{*}p < 0.05$; $^{**}p < 0.01$; $^{***}p < 0.001$
