# Supplementary material for: Breakdown of local information processing may underlie isoflurane anesthesia effects
Source: PLoS Comput Biol. 2017 Jun 1;13(6):e1005511. doi: 10.1371/journal.pcbi.1005511 (PMC5453425; doi:10.1371/journal.pcbi.1005511)
Supplement: S7 Table — Practical average running times for estimation of information-theoretic measures from one recording session and two recording sites or directions of interaction in animal 1 (see also supporting information S1 Text). (PDF) [file pcbi.1005511.s008.pdf]

**measure**	**toolbox/implementation**	**mean running time [min]**
TE_{SPO}	TRENTOOL/GPU-implementation	2235.93 (314.27 SD)
TE_{SPO}	TRENTOOL/CPU-implementation	1871.51 (926.56 SD)
AIS	TRENTOOL/CPU-implementation	2.83 (1.51 SD)
H	JIDT/CPU-implementation	1.55 (0.56 SD)

SD = standard deviation
