# Supplementary figures and images for: Breakdown of local information processing may underlie isoflurane anesthesia effects
Source: PLoS Comput Biol. 2017 Jun 1;13(6):e1005511. doi: 10.1371/journal.pcbi.1005511 (PMC5453425; doi:10.1371/journal.pcbi.1005511)

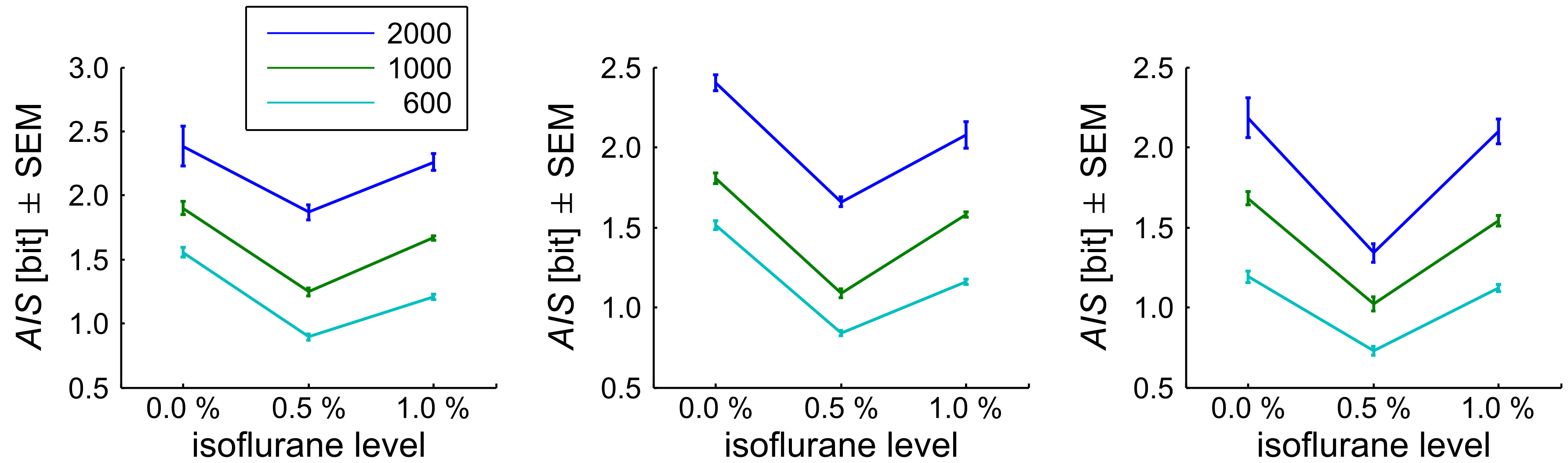

Supplement: S1 Fig — AIS estimates from data sampled at different rates. Data were chosen from three random recordings in animal 1 under three levels of Isoflurane. AIS was estimated from data with the sampling rate used for analysis in the present work (1000 Hz) and from data re-sampled at 2000 and 600 Hz respectively; note that qualitative results did not change due to re-sampling, but absolute estimates increased for higher sampling rates; the number of data points was held approximately constant by selecting a subset of trials for estimation such that the number of points entering the analysis was equal to the smallest number of points over all isoflurane levels. (TIF) [file pcbi.1005511.s009.tif]
