# Supplementary material for: Breakdown of local information processing may underlie isoflurane anesthesia effects
Source: PLoS Comput Biol. 2017 Jun 1;13(6):e1005511. doi: 10.1371/journal.pcbi.1005511 (PMC5453425; doi:10.1371/journal.pcbi.1005511)
Supplement: S1 Text — (PDF) [file pcbi.1005511.s010.pdf]

Theoretical and practical running time of nearest-neighbor based estimators

The KSG-estimator used in this work is computationally demanding, because as a nearest-neighbor based estimator it requires the execution of k-nearest-neighbor as well as range searches for all data points N. Hence, the algorithms used to perform these searches determine the asymptotic time complexity of the estimator. In this work, we used two different implementations published as part of the TRENTOOL toolbox: a CPU-based estimator for the estimation of TE_{SPO} from epochs or trials of data [1], and a GPU-based estimator for the estimation of TE_{SPO} from data pooled over epochs or trials [3]. We used the CPU-based work-flow for the epoch-wise estimation of TE_{SPO} and the GPU-based work-flow when testing for statistical significance of TE_{SPO} to maximize the number of points entering the estimation. The CPU-based estimator uses a neighbor-search algorithm that—depending on data-properties—requires for neighbor-searches on all N points at maximum $O(kN\log(N))$ time. The GPU-based estimator does not make use of fast data structures used for the CPU-based estimator, but uses a linear search which results in a worse time complexity of $O(dN^2)$. However, in the implementation used in this study [3] the linear neighbor search is performed in parallel over points and problem instances, which significantly improves the overall running time when using the estimator on multiple problem instances—a comparison of the running times of the serial CPU-algorithm and the parallel GPU-algorithm can be found in [3].

Additionally, we used the KL-estimator for entropy estimation. This estimator requires the execution of a k-nearest-neighbor search for all data points N—we used the estimator's implementation published as part of the JIDT toolbox [2], which has a time complexity of $O(kN\log(N))$.

We also measured practical running times of the estimators to provide a point of reference when planning similar analyses. Approximate, average running times for the estimation of AIS, TE_{SPO}, and H are presented as supporting information S7 Table. The presented running times include the estimation of each measure for both directions of interactions or recording site, for one recording; presented running times are averages over recordings. We measured the total time needed for estimation, including data preparation (e.g., the optimization of embedding parameters for the estimation of TE_{SPO}). All estimation procedures that did not require a GPU, were executed on a Intel(R) Xeon(R) CPU clocked at 2.90 GHz. The GPU-implementation of the TE_{SPO} estimator was run on a Intel(R) Xeon(R) CPU clocked at 2.00 GHz and a NVIDIA GeForce GTX TITAN. Both machines were running 64-bit Ubuntu Linux. Note that the GPU estimation was performed on 50 trials only, because here the computational demand was higher due to more data points entering one estimation of TE_{SPO} (pooled over epochs).

References

[1] Lindner M, Vicente R, Priesemann V, Wibral M. TRENTOOL: a Matlab open source toolbox to analyse information flow in time series data with transfer entropy. BMC Neurosci. 2011;12(1):119.

[2] Lizier JT. JIDT: an information-theoretic toolkit for studying the dynamics of complex systems. Front Robot AI. 2014;1(11).

[3] Wollstadt P, Martínez-Zarzuela M, Vicente R, Díaz-Pernas FJ, Wibral M. Efficient transfer entropy analysis of non-stationary neural time series. PLoS One. 2014;9(7):e102833.
